# Summer Rules

**Published:** 1870-08

**Authors:** 


### SUMMER RULES.

If you have walking and riding to do, ride first, because if you walk you may get over-heated and then, when you ride, you may be exposed to an open window or a draught of air while you are in a still position, to be followed by a chill, a pleurisy or lung fever, which is pneumonia.

If on any occasion you find yourself the least bit noticeably cool, or notice the very slightest disposition to a chill running along the back, as you value health and life, begin a brisk walk instantaneously and keep at it until perspiration begins to return; this will seldom fail to ward off a summer cold, which is more dangerous than. a cold taken in winter to all persons having the slightest tendency to consumption.

DRINKING WATER.

If very thirsty and warm, take but a swallow at a time, taking the glass from your lips with a dozen seconds between the swallows, then you will never fall dead while taking a drink, as many have; in this way half the amount of water will abundantly satisfy the thirst.

SODA WATER

is an agreeable beverage, but half a glass of cold water will better and more safely satisfy the thirst and costs nothing. Besides, in taking a glass of water, you stop when you feel you have had enough ; this you never do with a glass of soda, but keep on drinking after it is positively disagreeable; and you hate to stop, but drink on again, to prevent wasting it.

Never sleep in the day-time uncovered, in summer; it is always dangerous, even if it be but half an hour on a bed; a lace shawl is better for a covering than nothing. Many lie down for a few moments, especially ladies coming from a walk, visit, or shopping; they do not intend to go to sleep, just to rest a minute or two; but many times they do go to sleep and wake up with an indistinct chilly feeling, followed in many cases by serious illness.

When you reach home tired and weak, and may be accompanied with an indefinable feeling of sadness or depression, without being conscious of any adequate cause for it, don't take a drink of ice water, however thirsty, nor a glass of soda, nor a drink of wine, but a cup of hot tea, as hot as you can swallow comfortably ; the heat is of more value than the tea itself, but both combined, are of incalculable value; if you are sitting down to a meal in this tired condition of body, and mental depression, some hot tea, taken before anything is eaten, will rouse the circulation, exhilarate the stomach, rally the spirits, and make you a different, a better, and a happier man in less than ten minutes, because the increasing debility and downward progress of the system is arrested by the warmth of the water and the active quality of the tea, until strength begins to be imparted to the system from the food taken.

It is safe to cool oneself off by dabbling the hands in cold water; safer and more natural if the water is warm, by the rapid evaporation every time the hand is lifted out of the water. But it is positively dangerous to wash the face in cold water when much heated. It is not dangerous but pleasantly efficacious if warm water is used.

